# Multifaceted Interweaving Between Extracellular Matrix, Insulin Resistance, and Skeletal Muscle

**DOI:** 10.3390/cells7100148

**Published:** 2018-09-22

**Authors:** Khurshid Ahmad, Eun Ju Lee, Jun Sung Moon, So-Young Park, Inho Choi

**Affiliations:** 1 Department of Medical Biotechnology, Yeungnam University, Gyeongsan 38541, Korea; ahmadkhursheed2008@gmail.com (K.A.); gorapadoc0315@ynu.ac.kr (E.J.L.); 2 Department of Internal Medicine, College of Medicine, Yeungnam University, Daegu 42415, Korea; mjs7912@yu.ac.kr; 3 Department of Physiology, College of Medicine, Yeungnam University, Daegu 42415, Korea; sypark@med.yu.ac.kr

**Keywords:** extracellular matrix, insulin resistance, skeletal muscle, advanced glycation end products

## Abstract

The skeletal muscle provides movement and support to the skeleton, controls body temperature, and regulates the glucose level within the body. This is the core tissue of insulin-mediated glucose uptake via glucose transporter type 4 (GLUT4). The extracellular matrix (ECM) provides integrity and biochemical signals and plays an important role in myogenesis. In addition, it undergoes remodeling upon injury and/or repair, which is also related to insulin resistance (IR), a major cause of type 2 diabetes (T2DM). Altered signaling of integrin and ECM remodeling in diet-induced obesity is associated with IR. This review highlights the interweaving relationship between the ECM, IR, and skeletal muscle. In addition, the importance of the ECM in muscle integrity as well as cellular functions is explored. IR and skeletal muscle ECM remodeling has been discussed in clinical and nonclinical aspects. Furthermore, this review considers the role of ECM glycation and its effects on skeletal muscle homeostasis, concentrating on advanced glycation end products (AGEs) as an important risk factor for the development of IR. Understanding this complex interplay between the ECM, muscle, and IR may improve knowledge and help develop new ideas for novel therapeutics for several IR-associated myopathies and diabetes.

## 1. Introduction

The skeletal muscle constitutes nearly 40% of body mass and is primarily composed of myofiber, multinucleated contractile cells [1,2], and mainly provides mobility, protects and supports the skeleton, and regulates the body temperature and glucose homeostasis within the body [3]. The skeletal muscle is the core metabolic tissue for the insulin-stimulated uptake of glucose, accounting for approximately 85% through glucose transporter type 4 (GLUT4) [4,5]. Therefore, reduced responsiveness of skeletal muscle to insulin, that is, insulin resistance (IR), is a critical aspect of type 2 diabetes mellitus (T2DM) development [6,7].

The skeletal muscle has a diverse population of stem cells known as muscle stem (or satellite) cells (MSCs), which have a remarkable capability of muscle regeneration to the structural and functional integrity of the skeletal muscle [8]. Furthermore, MSCs can be transdifferentiated into adipocytes or osteocytes; hence, they are good models for intramuscular adipogenesis or osteogenesis studies [9].

**Figure 1:**
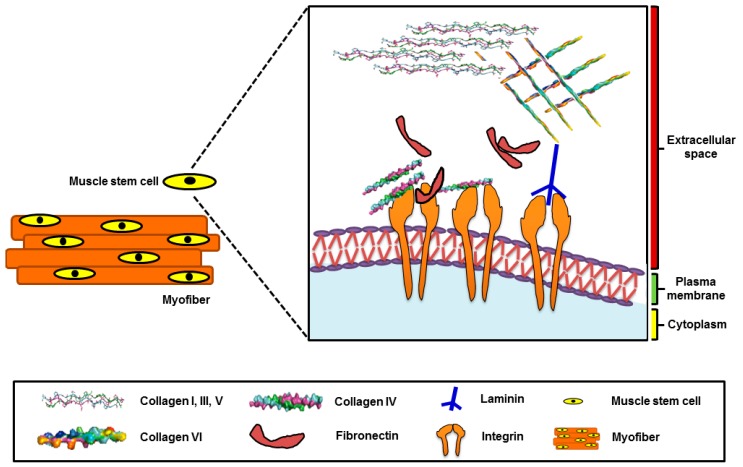
Muscle stem cells and the extracellular matrix (ECM) microenvironment.

The extracellular matrix (ECM) is composed of structural glycoproteins like collagens, laminin (s), and fibronectin (FN) bound to proteoglycans (PGs), which all help to maintain skeletal muscle integrity and provide constructional support. Furthermore, the ECM generates biochemical signals for myogenesis regulation [10]. The ECM also works as a growth factor modulator in the process of cell growth and is involved in various cell signaling processes [10,11]. Collagens are found abundantly in the ECM environment, and are essential for the mechanical support of tissues in addition to cell adhesion, wound healing, and differentiation. Types I, III, and IV collagens are expressed strongly in the skeletal muscle, in which types I and III are fibrillary, whereas type IV is expressed mainly in the basement membrane (BM). Furthermore, MSCs are located under the BM (Figure 1) [12]. The ECM, a highly dynamic structure, undergoes remodeling in a number of metabolic tissues because of injury and repair and is allied with diet-induced IR [13]. ECM remodeling and the altered expression of integrin is generally found in disease conditions. Several studies have examined whether ECM remodeling and altered signaling of the integrin receptor in the diet-induced condition is allied with IR [12,14]. In a study, the muscle-specific exclusion of integrin β1 in chow-fed mice resulted in reduced whole-body insulin sensitivity and reduced uptake of insulin-stimulated glucose during hyperinsulinemic-euglycemic clamp experiments (a gold-standard technique to measure insulin sensitivity) [13,15].

This review provides a brief introduction of skeletal muscle development, IR correlations, glycation, and diabetes. In addition, the relationship between ECM remodeling of the skeletal muscle and IR is discussed, and guidelines for the prevention and future directions to combat or minimize the manifestation of the disease conditions are proposed.

## 2. Extracellular Matrix

The ECM is a complex milieu of diverse structural molecules involved in structural support together with cellular signaling and tissue responses to diseases and injuries [16]. An organization of ECM molecules have their own distinct features and is responsible for the various biological activities [16,17]. A number of muscle-related genetically determined diseases are caused primarily by mutations in the ECM components and their receptors. For example, more than 150 ECM proteins interact with the adhesion site of the integrin receptor [18,19]. Maricela et al. performed a clinical study involving 66 patients with Duchenne/Becker muscular dystrophy (DMD/BMD), hyperinsulinemia, IR, and obesity. They suggested that an alteration in GLUT4 in muscle fibers from DMD/BMD patients might be involved in IR [20].

Different types of collagens are expressed in skeletal muscle with their specific role (Table 1). Collagens can be subdivided broadly into various subfamilies according to the sequence similarities and supramolecular assemblies they form, for example, fibrils, beaded filaments, anchoring fibrils, and networks [21]. Fibrillar collagens (types I, II, III, V, XI, XXIV, and XXVII) generally provide three-dimensional structures for tissues and organs [22]. These networks have mechanical strength and signaling functions by binding to the ECM components and cellular receptors. More than 90% of the collagens were found to be expressed in skeletal muscle and were composed mostly of collagen I, III, and IV [23,24]. Although collagen I and III encompasses fibrillar collagen, collagen IV is the most plentiful structural component of the BM [12,25]. Type I collagen is the most important and ample protein in the vertebrates (including humans), found ubiquitously in connective tissues, and is usually involved in the promotion of membrane proteinase activation, which leads to cellular migration and adhesion [26,27,28]. The packing and positioning of subfibrillar elements of the collagen structure characterize most of the biologically substantial aspects of the fibrillar collagen structure [26,28]. Seminal studies have shown that changes in the composition of the ECM (generally increased collagen) are a general characteristic of IR human skeletal muscle [12]. FN is a modular protein and an important structural element in the niche of MSCs that plays a vital role in the muscle regeneration process. The loss of FN from the niche affects many pathways and cellular mechanisms involved in MSC aging. In aged skeletal muscle, FN triggers adhesion signaling and functioning of MSCs. Treatment with FN is shown to restore the regenerative capacity of aged muscles [29]. In the previous studies, we explored the role of fibromodulin (FMOD) and matrix gla protein (MGP), the ECM proteins, involved in myoblast differentiation by regulating the interaction of myostatin (MSTN) with its receptor activin receptor type IIB (ACVRIIB). Involvement of FMOD and MGP in the regulation of myogenesis provides a clue for the development of novel therapeutics for the treatment of the different types of muscle diseases because it plays an important role by recruiting more MSCs to the sites of muscle injury [8,30,31,32].

ECM homeostasis is vital for normal functioning of a cell and for stable communication among cells, and a disruption of this homeostasis may have an adverse effect on the functioning of organ systems and promote many deadly diseases (e.g., fibrotic diseases and cancer) [40]. Furthermore, it has been reported on several occasions that the expression of several ECM proteins is altered during the onset of T2DM, and that these alterations change the ECM networks and consequently cell-to-cell and cell-to-ECM interactions [41]. The ECM components communicate with cells through cell surface receptors, and integrins are the most important. These receptors are composed of heterodimeric (alpha and beta) subunits, which interact with different types of the ECM ligands [13]. Seven alpha (α) subunits (α1, α3, α4, α5, α6, α7, and αv) in association with the β1 subunit are expressed in the skeletal muscle [13,42]. The integrin α7β1 expressed by MSCs is augmented in the myotendinous (MTJ) and neuromuscular junctions in the skeletal muscle.

### Muscle Stem Cells and Extracellular Matrix(ECM)

Muscle fibers or myofibers are the functional units of skeletal muscles, and are formed during embryogenesis when myoblasts fuse to form myotubes. MSCs are usually found in the quiescent phase and remain in this form until they are invoked by injury and exercise. Gentle injuries may initiate minimal proliferation, whereas major ones can recruit a larger number of MSCs and induce more proliferation earlier than differentiation [43]. Several factors regulate the activation of MSCs. Among them, some widely explored factors are muscle regulatory factors (MRFs: MYF5, MYOD: Myoblast determination protein 1, myogenin, etc.), hepatocyte growth factor (HGF), and neuronal nitric oxide synthase (NOS) [44]. MSCs are positioned between the sarcolemma (cell membrane) and BM (basal lamina; BL) of the muscle fibers, which are indicated as a well-equipped ‘niche’. The balance between the quiescent and activated form of MSCs is sustained mainly by this specific niche [45]. The ability of regeneration of the skeletal muscle is dependent primarily on the interaction between MSCs and their niche. The BL is encompassed by a network of ECM, which is connected directly to MSCs. Type IV collagen and laminin-2 are the main components of BL, and the concentration of these components diverges according to the function of the muscle fiber type. In addition to these two components, collagen, VI, perlecan, nidogen, FN, and other glycoproteins and PGs are the constituents of BL [45,46].

α7 and β1 integrins are typically expressed by MSCs to form a complex in BL and bind with laminin-2, though their expressions are dependent on the functions of activated MSCs [45]. Activated MSCs of mouse express β3 integrin, which probably forms a complex with αv integrin to produce the αv-β3 receptor for proteins having an exposed tripeptide of (Arg-Gly-Asp: RGD) ECM ligands, including FN, collagens, osteopontin, and laminins [47]. Another study showed that the activated MSCs induce confined remodeling of the ECM components and the deposition of laminin (α1 and α5) into the BL. The activation of AMP-activated protein kinase (AMPK), that is, the phosphorylated form, indorses glucose uptake and upsurges insulin sensitivity. The MSCs isolated from the injured muscles of diet-induced obese (DIO) mice determine the reduced AMPK activity and decreased regeneration [48].

## 3. Insulin Resistance in Skeletal Muscle

IR is defined as a decrease in the metabolic response of the skeletal muscle cell to insulin, which is a protruding feature of obesity and T2DM [49]. Insulin binding to the receptors in the cell membrane activates the signal transduction pathways, insulin receptor substrate (IRS)-1, phosphatidylinositide 3-kinases (PI3K), and AKT (protein kinase B, PKB), which mediates the insulin-stimulated glucose uptake via GLUT4 from the cytoplasm to the plasma membrane. Reduced insulin-induced activation of the signaling pathway and GLUT4 translocation lead to the development of insulin resistance and T2DM [50]. Although the precise mechanisms of IR are unclear, a robust relationship has been found between IR and obesity. Obesity is accompanied by increases in the lipid levels in the plasma and the accumulation of extra lipid, predominantly in the skeletal muscle and liver. The possible mechanisms through which obesity induces IR are increased fatty acid metabolites, oxidative stress, and inflammation, leading to suppression of the insulin signaling pathways. Another possible mechanism involved in the development of insulin resistance in obese subjects is a reduction of the vascular density in the skeletal muscle [51]. In addition, an interaction was reported between ankyrin-1(ANK1) and insulin receptor substrate-1 (IRS1) in skeletal muscle, and IRS1 is a key constituent of insulin signal transduction and arbitrates metabolic and mitogenic responses to insulin. ANK1 has been identified as a candidate gene for T2DM in skeletal muscle [52]. Furthermore, mutations in IRS1 protein are linked with IR, and in one study, the IRS1 gene was detected in T2DM patients that exhibited polymorphisms in over 11 amino acids [53].

Endoplasmic reticulum (ER) stress is associated with the relation between nonesterified fatty acid and IR, and eventually to the progression of T2DM. Direct contact between myotubes and palmitate acid induces ER stress [54]. Panzhinskiy et al. reported protein tyrosine phosphatase 1B (PTP1B), which is found on the ER membrane, acts as a negative regulator of insulin signaling activated by ER stress, and is essential for full activation of ER stress pathways that mediate IR in skeletal muscle [55]. Ijuin et al. demonstrated that skeletal muscle and kidney-enriched inositol polyphosphate phosphatase (SKIP), a key regulator of MSC differentiation, has a specific role in IR progression in skeletal muscle. Increased SKIP expression in the presence of ER stress was found to be significantly higher in the skeletal muscle of high-fat diet (HFD) and db/db mice than in wild-type controls [56].

IR in skeletal muscle is strongly linked with the lipid metabolism [57]. Increased levels of triglycerides (TGs) and fatty acids in the blood circulation and the augmented intracellular accumulation of several lipid intermediates are the hallmarks of this condition. Increased fatty acid uptake or a low rate of oxidation capacity in the presence of IR leads to higher concentrations of lipid intermediates in skeletal muscle cells. Furthermore, numerous studies have shown that an unbalanced diet or an HFD lead to accumulation of TGs and other byproducts of fatty acid oxidation due to incomplete or reduced oxidation of these fatty acids, which eventually causes IR in skeletal muscle [58,59]. It has also been reported that skeletal muscles of individuals with IR and/or T2DM are characterized by decreased oxidative capacity and mitochondrial contents and functions. Actually, oxidative capacity has been reported to better predict insulin sensitivity than intracellular TG or LC-CoA concentration in T2DM patients [60,61].

There are several other imperative factors regulating IR in skeletal muscle. Recently, a study reported that reduced signaling of p38 MAPK/JNK module instead of increased signaling in skeletal muscle endorses IR and metabolic syndrome [62]. Another study based on the Korean population with 14,807 participants (18–65 years of age) suggested that connotation of muscle mass with metabolic syndrome and IR is attenuated by high-fat mass [63].

## 4. The Extracellular Matrix (ECM) and Insulin Resistance

The ECM in the muscle plays a crucial role in the regulation of glucose homeostasis; a change in the composition of the ECM is a hallmark of IR muscle. Previous studies examined insulin-resistant muscle in diabetic and obese people and reported that collagen deposition was remarkably higher than that in normal people [64,65]. A study on healthy males who gained weight rapidly reported reduced insulin sensitivity and that a number of muscle ECM genes were upregulated. They reported that the reason for the weight gain was not due to local adipose tissues or any systemic inflammation, which eventually indicates the role of muscle ECM in the regulation of glucose homeostasis [13,35]. An imbalanced diet and over nutrition play contributory roles to the changes in the gene expression resulting in long-term obesity obstructions. Several studies have shown that the ECM genes (Type I, III, IV, V, and VI collagen), integrins (ITGBL1, ITGA4, and ITGA5) and matrix metalloproteases (MMP2 and MMP25) are upregulated in response to overfeeding. These findings suggest that ECM remodeling is coupled with the development of diet-induced IR, and is causative to the pathophysiology of T2DM [16,66]. MMPs are crucial for the degradation of the ECM components, and in particular, MMP 9 degrades collagen IV, a major constituent of basement membrane and an important factor in ECM remodeling. Kang et al. reported that the augmented deposition of collagen in diet-induced obese conditions is due in part to the reduced activity of MMP 9, and its genetic deletion increases the deposition of collagen in the muscle and impairs muscle IR in HFD mice [13,67]. It has also been suggested that the activation of growth factors (e.g., TGF-β1) by oxidative stress and inflammation causes ECM remodeling [68,69].

Recently, ECM remodeling in skeletal muscle has been reported in diabetic subjects. The skeletal muscles of diabetic rats showed a reduction in collagen integrity and altered normal triple helical structures [70]. Type I and III collagen levels are also elevated in skeletal muscle in diabetic patients [71] and a link between skeletal muscle ECM remodeling and IR has been proposed [16]. In one study, ECM-related gene expression was increased by a 48 h lipid infusion designed to induce IR [65]. Furthermore, in human subjects, short-term overfeeding induced IR and upregulation of the collagen I/III and MMP2 genes [66]. Although reports on the topic are limited, increasing evidence indicates that a fundamental relationship exists between skeletal muscle, ECM remodeling, and IR. Prior exposure of monkeys to whole-body radiation resulted in the ECM fibrosis and IR in skeletal muscle [71]. Furthermore, in the HFD-induced obese mice model, diet-induced IR was accompanied by increased deposition of hyaluronan/hyaluronic acid (HA) in skeletal muscle ECM, and subsequent long-term hyaluronidase treatment reversed IR by reducing HA levels [72].

The mechanisms of skeletal muscle ECM remodeling by induced IR is still unclear, but several hypotheses have been proposed. The most plausible hypothesis is a scarcity of microvasculature in the fibrotic ECM because a decrease in microvascular density supplies fewer nutrients and hormones to the skeletal muscle [67,71]. A decrease in capillary density has been proposed as one of the causes of IR in obese and older individuals [73,74,75,76]. Increased vascular density by angiopoietin-1 in high-fat-fed obese mice prevents the progression of IR in the skeletal muscle [77]. Furthermore, the prevention of muscle IR in the HFD mouse model by overexpressing catalase or by sildenafil (a phosphodiesterase 5a inhibitor) treatment reduced collagen I/III deposition and improved muscle vascularization [12,78,79]. Another hypothesis proposed for IR induction by fibrosis is that some components of remodeled ECM act to induce IR. In one study, integrin α (2) β(1)-null mice fed an HFD did not develop obesity-induced IR [12], and in another, reduction of HA in skeletal muscle ECM reversed HFD-induced IR [72].

Downstream integrin signaling through focal adhesion kinase (FAK) and integrin-linked kinase (ILK) might be a mechanistic connection between the muscle ECM and IR [13]. FAK is a tyrosine kinase with the properties of intracellular signaling, stabilization of cytoskeleton, and focal adhesion turnover, and is regulated by insulin receptors [80]. Bisht et al. reported that FAK is associated with the regulation of insulin action in the muscle because FAK tyrosine phosphorylation is reduced in the muscle from HFD rats [81]. Bisht et al. reported that the knockdown of FAK (in vivo siRNA-mediated) in chow-fed mice resulted in hyperinsulinemia, diminished glucose tolerance, and reduced insulin action [82].

ILK is an intracellular scaffolding protein that interacts with the cytoplasmic domains of b1, b2, and b3integrin [13]. ILK plays a critical role in muscle insulin action. Kang et al. reported that downstream integrin signaling through ILK is hazardous to the pathogenesis of IR. They showed that muscle-specific removal of ILK improves the muscle insulin sensitivity significantly in HFD-IR mice via the augmented phosphorylation of Akt [83].

Skeletal muscles are more susceptible to exercise-induced myofiber injury in the presence of T2DM, and T2DM-mediated changes in skeletal muscle depend on BM structure, and particularly on the activities of enzymes that regulate the synthesis of collagen. In a comparative microarray study of skeletal muscles, several types of collagen (type I, III, IV, V, VI, and XV) were downregulated and PGs (laminin-2, elastin, thrombospondin-1, and decorin), noncollagenous proteins, and connective tissue growth factor (CTGF) were upregulated in streptozotocin-induced diabetic mice compared to normal mice, and these changes eventually affected the basement membrane structure [84].

## 5. Glycation of Skeletal Muscle ECM

**Figure 2:**
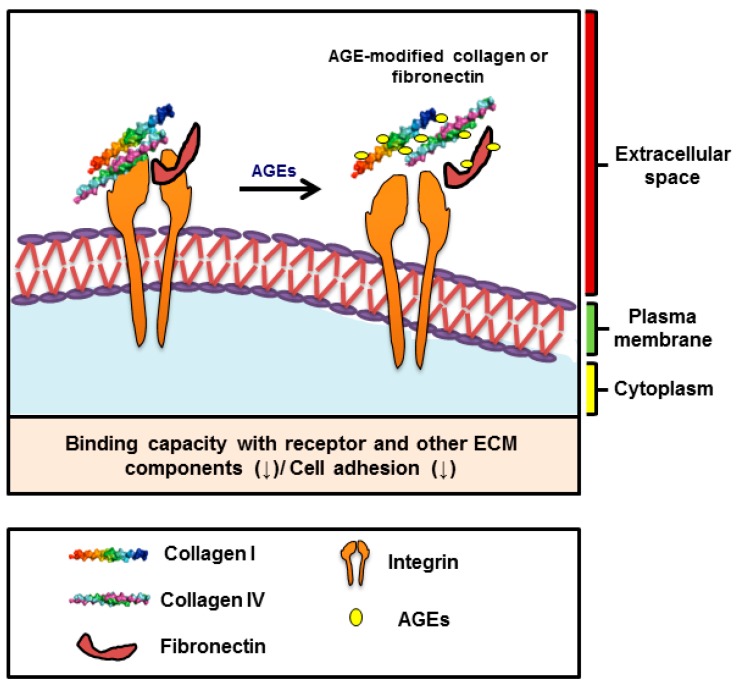
Modifications of ECM components by advanced glycation end products (AGEs) and their effects.

The nonenzymatic binding of a glucose molecule to proteins, lipids, or nucleic acids yields stable ‘Amadori products’, which undergo additional modifications to form advanced glycation end products (AGEs). AGEs are chemically heterogeneously modified molecules that form through the nonenzymatic glycation of proteins over an individual’s lifetime and have been implicated in a number of chronic diseases, such as diabetes [85]. Elevated levels of AGEs have been directly related to degrees of hyperglycemia, which underlies tissue damage and T2DM [86]. The formation of AGEs in diabetic patients is enhanced by high glucose concentrations in blood and is ‘impulsive’ and rather slow and predominantly affects proteins with comparatively long half-lives with exposed ‘lysine’ residues. The ECM proteins are usually long-lived and are latent targets of nonenzymatic glycation. Moreover, among the ECM proteins, collagens are highly vulnerable to glycation. Fibrillar collagens have exceptionally long half-lives, for example, type I collagen has half-lives of 1–2 years in bone and 10–15 years in skin, and type II collagen has a half-life exceeding 100 years in cartilage [87]. The glycation of fibrillar collagen is a surface phenomenon that produces cross-linkages, which subsequently modify matrix properties. The glycation of the ECM proteins causes structural alterations and disrupts binding affinities because it modifies the arginine residues of RGD and GFOGER motifs of major ECM components like FN and collagens. Furthermore, the intramolecular cross-links formed may confer proteolytic resistance, eventually leading to basement membrane coagulation [88]. In addition, AGE-mediated alterations in collagens I and IV affect the intensities of their interactions (binding capacity) with other components of the ECM as well as their capability to support cell adhesion. The interactions among the ECM components, such as collagen I and/or IV, FN, and heparin were found to be reduced by AGE-modification (Figure 2) [89,90,91].

AGEs are an important risk factor for the development of IR because they are discretely correlated with IR in healthy subjects [92]. Though insulin is not a target for AGE-modification as its half-life is short, in vivo and in vitro experiments (cultured in hyperglycemic conditions) have reported glycation sites on insulin [93]. Several AGE inhibitors, both natural and synthetic, have been identified. Our group recently investigated some potent AGE inhibitors, and during the study, we found silver nanoparticles (AgNPs) significantly and concentration-dependently inhibited AGE formation, which suggested they can be considered a candidate for the treatment of diabetes and diabetes-associated problems [85]. We also studied the roles of AGEs in muscle-related myopathies and found AGE production and subsequent receptor for advanced glycation endproducts (RAGE)-AGE binding hinders the myogenesis program. In addition, we found curcumin and gingerol both reduce the effects of AGEs on a muscle development program [94].

## 6. Insulin Resistance and Skeletal Muscle ECM Remodeling: Clinical Studies

Several clinical studies have shown that skeletal muscle ECM remodeling is closely associated with IR, obesity, and metabolic disorders. Richardson et al. first demonstrated that free fatty acid (FFA) markedly elevated ECM genes and collagen deposition in healthy human skeletal muscle [65]. These changes in the ECM composition are typically found in IR skeletal muscle, which also shows a robust increase in collagen content (types I and III) [64]. Furthermore, it is widely accepted that increased transforming growth factor beta (TGF-β) signaling results in ECM remodeling in IR skeletal muscle [95]. MSTN, a potent antianabolic regulator of muscle mass, has been reported to be upregulated in muscle myotubes and plasma in extremely obese subjects, and to be closely correlated with systemic IR [96]. On the other hand, it was also reported that Smad signaling (Suppressor of Mothers Against Decapentaplegic) was activated in association with a reduction in MYOD transcription, but it was observed that TGF-β1 and MSTN protein levels were not changed significantly [95]. A recent study reported failure of autophagy caused by overweight impaired myogenic differentiation in the elderly, but that MSTN expression did not change significantly [97]. Although studies in human subjects have produced conflicting results regarding ECM-related gene changes, in one study, Smad signaling was suggested to account for ECM remodeling, but neither TGF-β1 nor MSTN alone were found to be implicated in the atrophic effects on skeletal muscle [98].

In a study of overfeeding-induced weight gain (approximately 10%), skeletal remodeling of ECM genes was observed to dramatically increase in association with skeletal muscle inflammation, whereas slight change occurred in adipose tissue. Interestingly, in this study, there was no indication of systemic or local inflammation in adipose tissue despite the presence of IR [35]. Although the increase in body weight was small (as little as 3%) after short-term (4 weeks) overfeeding, the insulin sensitivity was markedly impaired, which was attributed to skeletal muscle ECM remodeling accompanied by increase in the mRNA expression of the ECM-related genes (*COL1α1, COL3α1*, *MMP2*). However, no significant changes were found in the expression of *MMP9*, *TIMP1*, *CD68*, and integrin. Thus, this small gain in body weight altered the expressions of genes related to ECM receptor interactions, such as focal adhesion and adherens junctions [66]. These results show skeletal muscle ECM remodeling plays a crucial role in the progression of obesity-induced IR and not in adipose tissue inflammation.

In a population-based study, adults aged over 65 years with elevated circulatory levels of carboxymethyl-lysine (CML) were found to be at a higher risk of impaired muscle quality (as determined by grip strength and gait speed testing), and this relationship remained significant after adjusting for risk factors [99,100]. In healthy middle-aged and older Japanese, AGE accumulation (measured by skin autofluorescence) was significantly correlated with lower muscle mass (skeletal muscle index) [101,102]. However, another small study showed that AGEs were not correlated with muscle mass but with lower limb muscle dysfunction in people with type 1 diabetes [103].

ECM remodeling of the skeletal muscle has attracted attention as a new therapeutic target for obesity and metabolic dysfunction. The skeletal muscle is highly adaptive to exercise, and regular exercise improves peripheral insulin sensitivity. Furthermore, both acute and long-term exercise activated significant amounts of genes in skeletal muscle (approximately 500 and 290, respectively), and ECM-related genes were also increased by the muscle’s adaptation mechanism to exercise (5% and 20%, respectively) [104,105]. In particular, serglycin, which is believed to be related to exercise adaptation by blocking serpinE1 (SERPINE1), was among several proteoglycans that were increased significantly by exercise [106]. *MSTN* gene expression was downregulated after acute and long-term exercise in IR skeletal muscles. Interestingly, *MSTN* expression increased in adipose tissues, but not in muscle cells, after 12 weeks of exercise, and this upregulation was found to be positively related to insulin sensitivity markers, indicating a tissue-specific effect [106].

Recently, attempts have been made to ameliorate IR using drugs targeting ECM remodeling. Bimagrumab (BYM338, Novartis) is a human monoclonal antibody that binds ActRII A and B, and thus interferes with their bindings to natural ligands, such as *MSTN*, growth Differentiation Factor11 (GDF11), and activin, which inhibit muscle growth [107]. A single dose of Bimagrumab showed decreased fat tissue volume by 7.9% and increased thigh muscle volume by around 2.7% in healthy lean subjects after 10 weeks. In IR subjects, Bimagrumab also increased skeletal muscle mass and reduced fat mass without causing body weight changes, and improved insulin sensitivity and metabolic statuses [108]. Although the results of human and animal studies often differ depending on experiment conditions and subject characteristics, ECM remodeling has become a new therapeutic target for metabolic disorders.

## 7. Concluding Remarks and Future Perspectives

The skeletal muscle is a crucial target for several metabolic syndromes, particularly T2DM, which is caused largely by IR. Insulin signaling is governed by several regulators, which include IRS1, GLUT4, and AKT as leading regulators. ER stress and fatty acid metabolism are the leading factors prompting IR in the skeletal muscle. In tissues, cellular interactions occur in the ECM, a three-dimensional network of polymeric biomolecules. The irregular expression of several ECM components, particularly collagen and MMPs, has been reported in skeletal muscle IR. Alterations in the ECM components perturb insulin signaling (inside-out and outside-in signaling) and alter the effects of insulin. ECM remodeling of skeletal muscle has only been recently proposed to be a mechanism of IR, and more evidence is required to prove the involvement of skeletal muscle ECM remodeling in IR. Future studies are needed to determine the mechanisms responsible for the manifestations of pathologic events in skeletal muscle, IR, and the ECM. An in-depth study of the connection between ECM remodeling of the skeletal muscle, the action mechanism of insulin, and integrin signaling will be a promising innovative line of research to develop novel therapeutics.

## Figures and Tables

**Table 1:** Major types of collagens in skeletal muscle ECM.

Collagen Types	Description	Expression During Diet-Induced IR	Reference
I	Abundantly found in endo-, peri-, and epimysium. Stimulate myogenic differentiation of stem cells.	↑	[16,33]
III	It is more consistently found between endomysium and epimysium.	↑	[34]
IV	Main component of basal lamina. Found to be 4 to 30-fold increase in skeletal muscle ECM mRNA levels	↑	[35]
V	Fibril-forming collagen and found to be increased in skeletal muscle ECM mRNA levels		[35]
VI	Found to be increased in skeletal muscle ECM mRNA levels		[35]
IX	Multiple-epiphyseal-dysplasia-related myopathy is caused due to mutation in collagen IX		[36]
XII	It is the largest member of the fibril-associated collagens with interrupted triple helix (FACIT) family. Important for muscle integrity.		[37]
XIV	A member of FACIT family and involved in muscle metabolism		[38]
XV	Extensively found in the basement membrane and a structural component vital to stabilizing the skeletal muscle		[39]
XVIII	Classified as multiplexins, bind with growth factors and other membranes of basement membrane glycoproteins.		[39]

## Abbreviations

MSCs	muscle stem cells
GLUT4	glucose transporter type 4
IR	insulin resistance
T2DM	type 2 diabetes mellitus
ECM	extracellular matrix
FN	fibronectin
MFRs	muscle regulatory factors
HGF	hepatocyte growth factor
NOS	neuronal nitric oxide synthase
BM	basement membrane
BL	basal lamina
AMPK	AMP-activated protein kinase
GWAS	genome-wide association studies
ANK1	ankyrin-1
IRS	insulin receptor substrate-1
PFKM	phosphofructokinase
ER	endoplasmic reticulum
IRS	insulin receptor substrate
IRE-1	inositol-requiring enzyme 1
TRB3	ER stress upraises tribbles 3
HFD	high-fat diet
PTP1B	protein tyrosine phosphatase 1B
SKIP	inositol polyphosphate phosphatase
TGs	triglycerides
MMP	matrix metalloproteases
FMOD	fibromodulin
MGP	matrix gla protein
MSTN	myostatin
ACVRIIB	activin receptor type II B
CTGF	connective tissue growth factor
MTJ	myotendinous
HA	hyaluronan
TGF	transforming growth factor
AGEs	advanced glycation end products
